# Prophylactic vs therapeutic blood transfusions impact on pregnant sickle cell patients

**DOI:** 10.12669/pjms.41.1.9783

**Published:** 2025-01

**Authors:** Iffat Imran, Imran Nazir, Rakan Tariq J Al Rfaai, Khalid Khalil

**Affiliations:** 1 Iffat Imran Consultant Gynecologist, College of Medicine, Taif University, Taif, Saudi Arabia; 2 Imran Nazir University Medical and Dental College, Faisalabad; 3 Rakan Tariq J Al Rfaai Security Forces Hospital Makkah, Makkah, Saudi Arabia; 4 Khalid Khalil Security Forces Hospital Makkah, Makkah, Saudi Arabia

**Keywords:** Pregnancy, Sickle cell disease, Blood transfusion

## Abstract

**Objective:**

To observe the fetomaternal outcome of therapeutic versus prophylactic blood transfusions in patients with sickle cell disease (SCD) during pregnancy.

**Method:**

This single-center retrospective observational study was conducted on consecutive pregnant women with SCD between January 2018 and December 2020. All the pregnant women with SCD were included in this study. Sickler women carrying sickle cell traits (HbAS, HbAC), multiple pregnancy, or other medical diseases were excluded from the study. We used non-random, convenience sampling and collected their demographic details, medical history, course of SCD before & during pregnancy, maternal/fetal outcome, laboratory parameters, and other information using the questionnaire. Patients were sub-grouped into three according to blood transfusion categories (therapeutic, prophylactic, or not transfused) during the pregnancy. Descriptive data were represented as numbers and mean ± SD values. The medical and obstetrical complications and fetomaternal outcomes among the three groups were analyzed by Chi-square/Fisher’s Extract test.

**Results:**

The study included 62 patients, 37 were in the therapeutic group, 11 were in the prophylactic group, and 14 were in the non-transfusion group. Hemolytic crises, painful crises, acute chest syndrome, pregnancy-induced hypertension (PIH), preeclampsia, preterm births, intrauterine growth retardation (IUGR), and neonatal ICU admission were significantly lower among the prophylactic group (P = 0.000, p = 0.000, p = 0.001, p = 0.002, p = 0.009, p = 0.007, p =0.001 & p= 0.016 respectively) compared with other two groups.

**Conclusions:**

Our study demonstrates that prophylactic blood transfusion in SCD positively alters the course of pregnancy by reducing fetomaternal complications.

## INTRODUCTION

SCD is a qualitative hematological disorder characterized by autosomal recessive inheritance. Due to this condition, the β subunit of hemoglobin synthesis is affected, producing abnormal sickle-shaped RBCs that can impede blood flow in smaller blood vessels, vaso-occlusion, ischemia, acute pain episodes, and chronic hemolytic anemia.^1^,^2^ SCD is a group of different genotypes. Homozygous form (HbSS) or other abnormal hemoglobins such as hemoglobin C (HbSC), and beta-thalassemia (HbSB thalassemia) are a few examples. Sickle cell disease presents severity in full swing in homozygous sicklers (HbSS), traits (HbAS, HbAC) usually have a benign course.^3^ Apart from SCD, multiple other factors cause anemia during pregnancy and worsen the outcome.^4^,^5^

Around 300,000 to 400,000 affected infants are reported to be born globally annually.^6^ In the KSA, the SCD prevalence is said to be 2.6%, and the 2-27% population is found to carry different traits.^7^ In the recent era with the advancement of understanding the pathophysiology and management of the disease, Sticklers do reach the reproductive age and have successful pregnancies.^8^ Pregnancy carries a grim load on SCD, owing to its tremendous hemodynamic alteration and stress on hematopoiesis, resulting in significant maternal and fetal morbidity as well as mortality.^9^

At present, the available management options include blood transfusion, hydroxycarbamide, and stem cell transplantation.^2^ Blood transfusion remains one of the fundamental supports, improving hemoglobin levels as well as reducing vaso-occlusion in pregnancy.^7^,^8^ The role of blood transfusions during pregnancy has been controversial. How should this be considered in sickle cell pregnancies? Prophylactic (preventative) or selective (therapeutic)?^10^ Selective (therapeutic) blood transfusion is indicated in severe anemia, painful sickle crises, acute chest syndrome, malaria infection, and sepsis.^11^

On the other hand, prophylactic blood transfusion is given to asymptomatic pregnant women with SCD to optimize their oxygen-carrying capacity of blood and reduce possible complications because of abnormalities related to red cells and chronic anemia. The aim is to attain a suitable Hb level ie around 10 g/dl and reduce the amount of HbS to less than 40% of the total.^11^ Fortin, Hopewell, & Estcourt provided a comprehensive review of relevant studies on blood transfusion to treat or prevent complications in sickle cell disease.^12^

Therefore, the selection of either prophylactic or therapeutic blood transfusion modality during pregnancy remains a dilemma. Because of the high prevalence of SCD and limited local data on this clinical problem, this study was designed to explore the effect of prophylactic or on-demand (therapeutic) blood transfusions in reducing maternal and fetal complications associated with sickle cell disease, specifically in Saudi Arabia.

## METHOD

This retrospective observational study was carried out in Security Forces Hospital Makkah (SFHM), Saudi Arabia on SCD pregnant patients, to determine fetomaternal outcomes in different blood transfusion modalities during pregnancy.

### Ethical Approval:

The first step was to get the ethical approval by SFHM IRB registered at the National BioMedical Ethics Committee, King Abdulaziz City for Science and Technology with IRB# 0353-22042 on April 29^th^, 2020.

### Inclusion and Exclusion Criteria:

All Sickle cell disease (SCD) pregnant women were included except sickle cell trait (carrier) patients having heterozygous hemoglobin S and C (HbAS, HbAC). Also, SCD patients having multiple pregnancies were excluded. Three years of electronic medical records between January 2018 and December 2020 were retrieved. All cases were included by convenient sampling technique, and their demographic data, detailed history of SCD management & course of the disease before & during the pregnancy, maternal/fetal outcome, laboratory parameters, and other details according to the questionnaire were collected. Patients were sub-grouped into three groups according to blood transfusion treatment modalities (therapeutic, prophylactic, or no transfusion given) during the pregnancy.

The first group comprised patients who received therapeutic blood transfusions; either indicated by some obstetric complication or acute SCD- complications like vaso-occlusive, hemolytic crises, acute chest syndrome, or stroke. The second group was those who received blood transfusions to optimize hemoglobin levels to the required level i.e. the prophylactic blood transfusion group. The third group received no transfusion during pregnancy. Comparing maternal and fetal mortality across the three treatment groups of patients was one of the primary measured outcomes. The secondary maternal measured outcomes were sickle cell crisis, total units of blood transfused, postpartum hemorrhage, urinary tract infection (UTI), pre-eclampsia, preterm birth, placenta abruption, and wound infections.

### Statistical Analysis:

Fetal/neonatal measured outcomes were intrauterine growth restriction, hemolytic disease of the newborn, prematurity and its sequelae, and low birth weight. Statistical analysis was done using SPSS version 27. Data for categorical variables were expressed as numbers and percentages; data for continuous variables were expressed as numbers, means ± standard deviation (SD). Maternal and fetal complications were compared among the groups using the Chi-square/Fisher’s Exact test depending upon the number of affected cases. Significant results were defined as ones having a P-value less than 0.05.

## RESULTS

This study included a total of sixty-two pregnant women with SCD. The mean maternal age was 28.56 ± 4.881 years, with the majority ie 85.5% from 20-35 years. Multipara were 54.8% and 45.2% were nullipara. It was found that 62.9% of the patients were on frequent or occasional blood transfusions before being pregnant. RBC transfusions were administered to 51 (82.25%) of the study participants for either therapeutic or prophylactic purposes. Therapeutic RBC transfusion was offered to 37 (59.7%) patients. The majority required it because of medical indications/complications; a minor group was those of obstetric reasons like post-partum hemorrhage (PPH). A prophylactic regime was observed to be offered to 14 (22.58%) patients in this study. Further, eight (12.9%) were those who were on chronic blood transfusion before conception, so the therapy was maintained with established chronic transfusion protocols including precaution of hemolytic and vaso-occlusive measures. The other six (9.67%) participants had asymptomatic anemia and were offered the RBC transfusion to achieve the target hemoglobin. The 3rd group comprised eleven (17.74%) sickle cell pregnant patients who did not receive any RBC transfusion.

**Fig.1:**
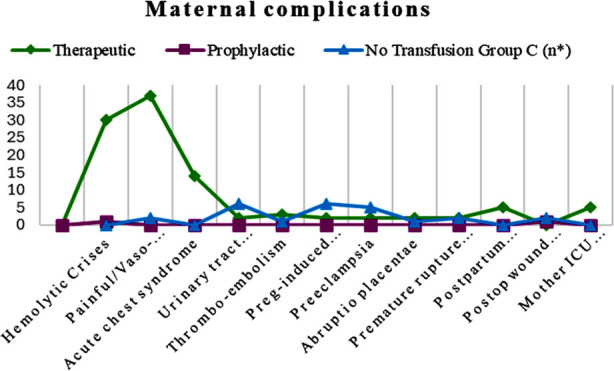
Comparison of maternal complications.

**Fig.2:**
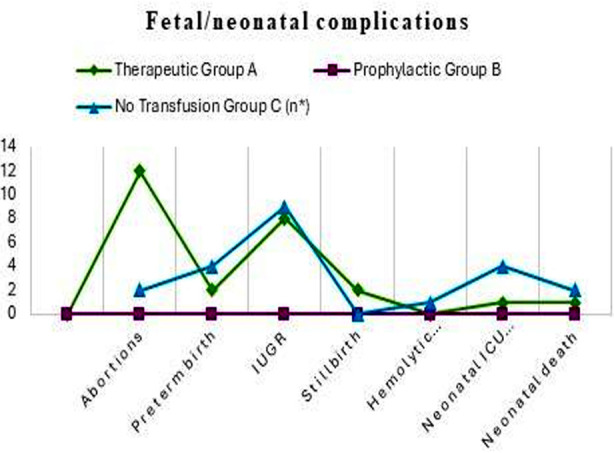
Comparison of feta and neonatal complications.

Anemia was observed in all 62 (100%) study participants with a mean value of 7.4 gm/dl and SD ± 1.348. No maternal death was observed in our study. The patients in the therapeutic group underwent frequent blood transfusions. Fourteen patients received 1-3 transfusions, ten patients received 4-6, seven received 7-9, and six patients received more than 10 blood transfusions throughout the course of pregnancy. The prophylactic group patients underwent significantly fewer transfusions, with nine patients receiving 1-3 and two patients receiving 4-6 blood transfusions, with a p-value of 0.000. The timing and indications of blood transfusions for either group was based on clinical assessments. Regarding maternal complications, vaso-occlusive crises were the most frequent complications observed. Other major maternal obstetric and non-obstetric complications are expressed in Table-I and comparisons among the three groups are presented in Fig.1. In this study, Intrauterine growth restriction (IUGR) affected 17 (29.8%) of the pregnancies of therapeutic and no transfusion group participants. Other fetal complications are listed in Table II and Fig.2.

**Table-I:** Maternal outcome in three groups of treatment modality.

Maternal Complications	Frequency & Percentage N*	Therapeutic Group A n*	Prophylactic Group B n*	No Transfusion Group C n*	P-value
Hemolytic Crises	31	30	1	0	0.000
Painful/Vaso-occlusive crises	39	37	0	2	0.000
Acute chest syndrome	14	14	0	0	0.001
Urinary tract infection	8	2	0	6	0.002
Thrombo-embolism	4	3	0	1	1.000
Preg-induced hypertension	8	2	0	6	0.002
Preeclampsia	7	2	0	5	0.009
Abruptio placentae	3	2	0	1	1.000
Premature rupture of membrane	4	2	0	2	0.350
Postpartum hemorrhage	5	5	0	0	0.268
Postop wound infections	3	0	1	2	0.061
Mother ICU admission	5	5	0	0	0.268

* Number of the total number of cases.

**Table-II:** Fetal outcome in three groups of treatment modality.

Fetal Complications	Frequency & Percentage N & %*	Therapeutic Group A n & %*	Prophylactic Group B n & %*	No Transfusion Group C n & %*	P-value
Abortions	14	12	0	2	0.054
Preterm birth	6	2	0	4	0.007
IUGR	17	8	0	9	0.001
Stillbirth	2	2	0	0	0.174
Hemolytic disease of the newborn	1	0	0	1	0.403
Neonatal ICU admission	5	1	0	4	0.016
Neonatal death	3	1	0	2	0.204

* Number of the total number of cases.

As demonstrated by Fisher’s exact test, most of the SCD complications, vaso-occlusive crises, hemolytic crises, acute chest syndrome, urinary tract infections (UTI), PIH, and pre-eclampsia showed a statistically significant less frequency (p= 0.000, 0.000, 0.001, 0.002, 0.002, and 0.009, respectively) with the prophylactic blood transfusion group compared to the therapeutic blood transfusion group. Similarly, fetal complications like prematurity & IUGR also had a statistically significant correlation with the prophylactic blood transfusion group as compared to the therapeutic blood transfusion group.

Therapeutic and prophylactic blood transfusion groups were not significantly different in terms of SCD-related thromboembolic events preterm labor, abruptio placentae, PROM, PPH, wound infection, stillbirth, neonatal ICU admission, hemolytic disease of newborn, neonatal death, and maternal ICU admission. Maternal and fetal complications frequencies in each group of blood transfusion (BT) modalities and P values are mentioned in Tables-I & II.

## DISCUSSION

Considering pregnancy demands on the steady condition of chronic destruction of red blood cells, anemia, and hypoxia, a substantial fraction of pregnant SCD women may require blood transfusion therapy during pregnancies.^13^ Therefore, the main concern of the current study was whether this intervention should be preventive or on-demand (therapeutic).

We observed maternal complications, such as hemolytic crises, vaso-occlusive crises, acute chest syndrome, UTI, PIH, preeclampsia, and abortions, which were significantly less frequent in the prophylactic blood transfusion (BT) group than in the therapeutic and non-transfusion groups. These findings are compatible with the results reported by multiple international studies.^14^-^16^ Furthermore, a recent meta-analysis of 15 cohort studies documented reduced vaso-occlusive events, maternal mortality, and perinatal complications.^17^ However, a few studies found no difference in maternal morbidity and mortality related to either blood transfusion approach.^18^,^19^

Our study documents significantly better fetal and neonatal outcomes, for example, no abortions and neonatal ICU admissions with prophylactic BT. Multiple studies have shown that prophylactic RBC transfusion in pregnant women with SCD reduces perinatal mortality, neonatal death, and adverse pregnancy outcomes, supporting the potential benefits of transfusions during pregnancy.^15^-^17^,^19^-^21^ However, Babasola and Olufemi found no difference in perinatal outcomes.^19^

The prophylactic blood transfusion has a dual beneficial effect; this regime attains a sufficient Hb level to comply with the pregnancy needs and reduces the amount of HbS decreasing the chances of SCD-related vaso-occlusive complications.^13^ Still the clinical assessment of the SCD patient, co-morbidities, past treatment record, and complications history are important factors in deciding the blood transfusion modality during pregnancy. Secondly, different guidelines addressed this issue differently; American College Obstetricians & Gynecology (ACOG) guidelines recommend just keeping HbS to around 40% and total hemoglobin concentration to around 10gm/dl.^22^ The British Society for Hematology recommends that pregnant women with severe SCD and women with current or previous obstetric, medical, or fetal problems be given prophylactic transfusions.^22^ Prophylactic transfusion during pregnancy is not recommended according to current UK standards.^10^ Owing to the lack of consensus on the ideal transfusion regimen and observational study designs of previous research, it is challenging to assess and compare the sole effects of prophylactic blood transfusion on pregnancy outcomes.

Also, it has been reported that transfusions can prevent adverse outcomes; however, the extensive use of red blood cells is also associated with risks. Risks related to transfusions include infections, acute transfusion reactions, and iron overload.^21^ Moreover, transfusion carries the risk of alloimmunization, which can complicate future transfusions. Kuriri etal. reported 16.7% alloimmunizations and 5.3% autoimmunizations among SCD patients in eastern Saudi Arabia.^23^ Ministry of Health Saudi Arabia SCD highlights patients’ awareness that sickle cell patients typically do not receive blood transfusions, but the issue may be discussed with them if necessary.^24^

Despite being used as a preventative measure, blood transfusions may or may not help in pregnancy. For instance, 11.6% of pregnant women who received preventative blood transfusions nevertheless required emergency blood transfusions for severe anemia or other complications.^25^

Owing to the lack of consensus on the ideal transfusion regimen among clinicians and hospitals, it is challenging to assess and compare the sole effects of prophylactic blood transfusion on pregnancy outcomes. This is partly because observational studies, which have inherent limits in the creation of relevant policies, were mostly used to determine the usefulness of the practice.

## CONCLUSION

SCD is a chronic hematological disorder that requires continuous care throughout pregnancy. A careful prophylactic blood transfusion approach not only ensures avoidance of complications but also improves overall maternal and neonatal morbidity. A multidisciplinary team must carefully select SCD patients to decide on an early pregnancy transfusion plan, which should include indications, goals, and monitoring protocols to improve outcomes for these high-risk pregnancies.
